# Hyperbaric Oxygen Therapy as an Adjunctive Treatment for Pediatric Fournier’s Gangrene

**DOI:** 10.7759/cureus.117815

**Published:** 2026-10-12

**Authors:** Zineb Elkhanfari, Soukaina Ait Hmadouche, Sara Babour, Abdelilah Radi, Rachid Abilkassem

**Affiliations:** 1 Pediatrics, Centre Hospitalier Ibn Sina, Rabat, MAR; 2 Pediatrics, Hôpital Militaire Avicenne, Rabat, MAR

**Keywords:** fournier’s gangrene, hyperbaric oxygen therapy, klebsiella pneumoniae, necrotizing fasciitis, neurogenic bladder, pediatric patient

## Abstract

Fournier’s gangrene (FG) is a rapidly progressive necrotizing soft-tissue infection involving the perineal and genital regions. Although predominantly described in adults, it is rare in children and may be associated with substantial morbidity and mortality. Early recognition, prompt broad-spectrum antimicrobial therapy, and urgent surgical debridement remain the cornerstone of treatment. Hyperbaric oxygen therapy (HBOT) has been proposed as an adjunctive treatment because of its ability to increase tissue oxygenation and potentially promote wound healing in ischemic and infected tissues. However, evidence supporting its use in pediatric FG remains limited. We report the case of a 14-year-old boy with a neurogenic bladder, bilateral ureterohydronephrosis, and chronic kidney disease who developed Fournier’s gangrene associated with a chronic urinary catheter and infection with ceftriaxone-resistant *Klebsiella pneumoniae (K. pneumoniae)*. The patient presented with fever and acute scrotal inflammatory changes. Computed tomography demonstrated extensive inflammatory changes of the peripenile and periscrotal soft tissues with multiple intratissue gas foci, consistent with FG. Emergency surgical exploration and extensive debridement were performed, followed by targeted antimicrobial therapy with imipenem and amikacin. HBOT was subsequently administered as an adjunctive treatment, with a total of 10 sessions, in addition to intensive local wound care. Clinical improvement was observed, allowing subsequent surgical revision and closure of the scrotal wound. This case highlights the importance of early recognition and multidisciplinary management of pediatric FG and illustrates the potential role of HBOT as an adjunct to surgical and antimicrobial treatment in selected pediatric patients.

## Introduction

Fournier’s gangrene (FG) is a severe and rapidly progressive necrotizing soft-tissue infection involving the perineal, genital, and perianal regions. First described by Jean-Alfred Fournier in 1883, it is characterized by infection spreading along fascial planes, microvascular thrombosis, tissue ischemia, and progressive necrosis [1,2]. Although FG predominantly affects adults, it is uncommon in children; however, historical pediatric reviews and more recent series confirm it can occur across the entire pediatric age range [3-6].

The epidemiology and predisposing conditions of pediatric FG differ from those typically encountered in adults. Reported pediatric associations include prematurity, omphalitis, diaper dermatitis, circumcision, perineal or anorectal infection, trauma, varicella, immunocompromising conditions, and congenital or acquired genitourinary abnormalities [4-8]. Genitourinary anomalies and urethral or urinary tract manipulation are particularly relevant because disruption of local barriers may provide a portal of entry for infection [6,9]. In the present patient, neurogenic bladder, bilateral ureterohydronephrosis, chronic kidney disease, and repeated urinary catheter replacement represented clinically plausible predisposing factors.

The early clinical picture may be deceptively nonspecific, with fever, pain, erythema, and scrotal swelling mimicking epididymo-orchitis, abscess, or other causes of an acute scrotum. Because progression can be rapid, a high index of suspicion is required. Ultrasonography may identify scrotal or testicular abnormalities, whereas computed tomography can delineate the extent of fascial and soft-tissue involvement and demonstrate findings such as fat stranding, fascial thickening, fluid collections, and soft-tissue gas [2,6]. Imaging can support diagnosis and operative planning in stable patients, but it should not postpone surgical exploration when necrotizing infection is strongly suspected [2,6].

Management is a surgical emergency and requires prompt resuscitation, broad-spectrum antimicrobial therapy, and urgent debridement of all devitalized tissue, followed by close reassessment and meticulous wound care [2,6,10]. Repeated debridement may be required when necrosis progresses beyond the initially apparent margins. Pediatric reports emphasize that favorable outcomes depend on early recognition and coordinated multidisciplinary care, while reconstruction or wound closure must be individualized according to the extent of tissue loss and local evolution [4-6,11,12].

Hyperbaric oxygen therapy (HBOT) has been proposed as an adjunct to standard treatment. Exposure to 100% oxygen at increased atmospheric pressure raises dissolved oxygen levels and tissue oxygen tension, which may improve oxygen delivery to ischemic tissue, support leukocyte oxidative killing, and promote angiogenesis, collagen synthesis, and wound healing [13,14]. However, the evidence in FG is derived mainly from retrospective observational studies. Meta-analyses in predominantly adult populations have reported an association between HBOT and lower mortality, without consistent reductions in hospital stay or the number of debridements [14,15]. HBOT therefore remains an adjunct and must never delay surgical source control. Pediatric experience is particularly limited [6]. We report a 14-year-old boy with neurogenic bladder and chronic urinary catheterization who developed ceftriaxone-resistant *Klebsiella pneumoniae (K. pneumoniae)*-associated FG and was managed with urgent debridement, targeted antimicrobial therapy, intensive wound care, and adjunctive HBOT.

## Case presentation

A 14-year-old boy was admitted to the pediatric emergency department with acute scrotal erythema associated with fever. His medical history was significant for bilateral ureterohydronephrosis and chronic kidney disease secondary to a neurogenic bladder. He was under regular urological follow-up and required chronic urinary catheterization, with replacement of a silicone indwelling urinary catheter every week.

On admission, the patient presented with fever and acute inflammatory changes involving the scrotal region. Laboratory investigations revealed a marked systemic inflammatory response and urinary infection (Table 1).

**Table 1:** Laboratory results at admission WBC: white blood cell count; CRP: C-reactive protein; ECBU: urine leukocytes The laboratory findings demonstrate a severe systemic inflammatory response, with marked leukocytosis (20,200/mm³) and neutrophil predominance (84%), associated with a markedly elevated CRP level (266 mg/L). Urinalysis shows significant leukocyturia (300 leukocytes/mm³), and urine culture is positive for *Klebsiella pneumoniae*, supporting a urinary bacterial infection

Parameter	Result	Unit	Reference range	Interpretation
WBC	20,200	/mm³	4,000-10,000 /mm³	High
Neutrophils	84	%	40-75%	High
CRP	266	mg/L	<5 mg/L	Markedly high
Urea	1.72	g/L	0.15-0.45 g/L	High
Creatinine	58	mg/l	0.5-1.3 mg/dL	Extremely high
ECBU	300	/mm³	<10 /mm³	High
Urine culture	Klebsiella pneumoniae	-	No significant bacterial growth	Positive

Initial scrotal ultrasonography showed findings suggestive of epididymo-orchitis associated with funiculitis and pyocele. Computed tomography of the pelvis demonstrated marked thickening of the peripenile and periscrotal soft tissues, associated with infiltration of the surrounding fat planes and multiple foci of gas within the soft tissues. These findings were highly suggestive of necrotizing soft-tissue infection and confirmed the diagnosis of FG (Figure 1).

**Figure 1:**
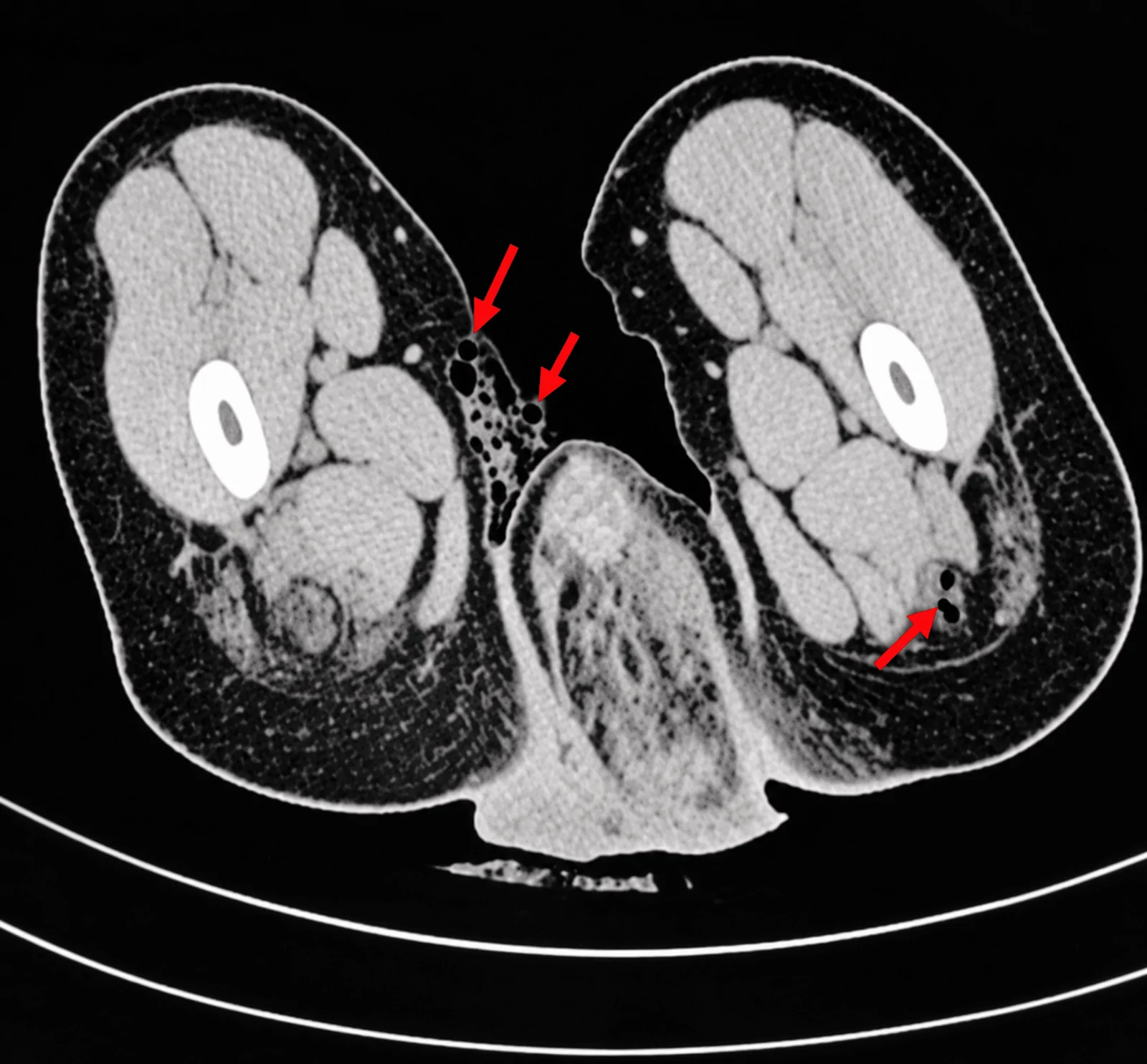
Contrast-enhanced computed tomography findings of Fournier's gangrene Pelvic computed tomography demonstrated marked soft tissues, associated with inflammatory infiltration of the surrounding fat planes and multiple foci of soft tissue gas

The patient underwent emergency surgical exploration and drainage under general anesthesia. A midline scrotal incision revealed extensive purulent material and necrotic tissue. Extensive debridement of all visibly devitalized tissues was performed until viable tissue was identified. The wound was left open to allow adequate drainage and facilitate postoperative wound assessment.

Samples of the scrotal purulent material were obtained intraoperatively for microbiological analysis. Culture grew *K. pneumoniae*. Antimicrobial susceptibility testing demonstrated resistance to ceftriaxone. Antibiotic therapy was therefore modified according to the susceptibility profile, and treatment with imipenem and amikacin was initiated.

Postoperatively, the patient received intensive local wound management with daily wound care and copious irrigation. Given the extent of the soft-tissue infection and the need to optimize tissue oxygenation and wound healing, adjunctive HBOT was initiated following surgical debridement. The patient received a total of 10 HBOT sessions, each lasting 60 minutes, administered once daily. HBOT was initiated two weeks after the surgical debridement.

The patient demonstrated progressive clinical improvement following combined surgical, antimicrobial, wound-care, and HBOT management. The local inflammatory changes regressed, and the wound showed satisfactory evolution without evidence of progressive necrosis. A subsequent surgical revision was performed after completion of the HBOT sessions, allowing closure of the scrotal incision (Figure 2).

**Figure 2:**
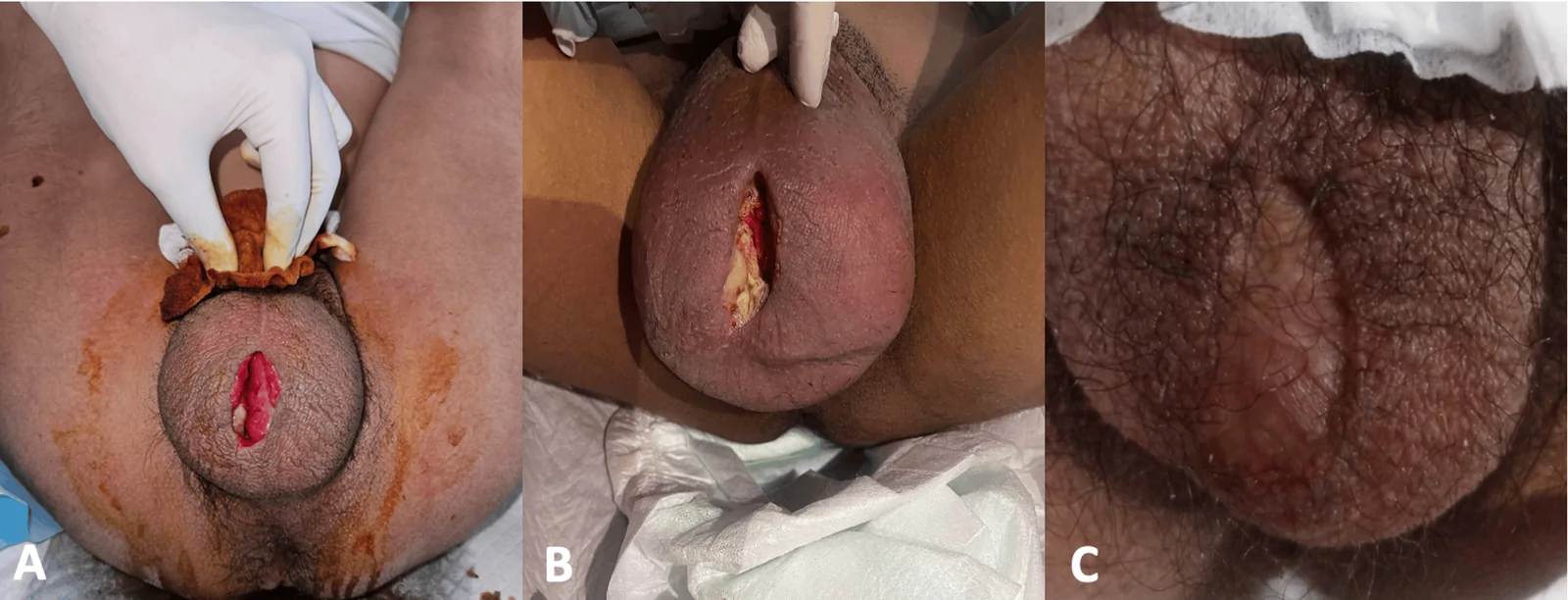
Clinical evolution of the scrotal surgical wound following debridement, HBOT, and subsequent surgical closure HBOT: hyperbaric oxygen therapy (A) Postoperative wound immediately after debridement; (B) wound during HBOT/local wound care; (C) wound after surgical revision and closure; (D) final follow-up appearance

The postoperative course was favorable, with progressive wound healing and no documented recurrence of infection during the available follow-up period. At the last clinical assessment, the patient was clinically stable, with satisfactory healing of the surgical wound.

## Discussion

FG is a life-threatening necrotizing soft-tissue infection in which rapid spread along fascial planes, microvascular thrombosis, ischemia, and tissue necrosis can lead to severe local destruction and systemic toxicity [2,6]. Although it remains rare in childhood, the pediatric literature now spans historical reviews, small case series, and recent reports, underscoring the heterogeneity of triggers and the need for early recognition [3-6].

Predisposing factors in children differ from the classic adult profile. Prematurity, diaper dermatitis, omphalitis, varicella, perineal infection, trauma, postoperative states, and congenital genitourinary or gastrointestinal abnormalities have all been described [4-8,12]. A particularly relevant comparison for our patient is the published pediatric experience linking FG with congenital genitourinary anomalies or urethral manipulation [6,9]. Our patient had a neurogenic bladder, bilateral ureterohydronephrosis, chronic kidney disease, and weekly replacement of an indwelling urinary catheter. Although a direct causal relationship cannot be proven, chronic urological disease and repeated instrumentation provide a biologically plausible route for bacterial entry and may have increased susceptibility to severe perineal infection.

The initial presentation in our patient also illustrates an important diagnostic difficulty. Scrotal erythema, swelling, fever, and pain may initially resemble epididymo-orchitis, pyocele, abscess, or other causes of an acute scrotum. Pediatric reviews emphasize that the diagnosis is primarily clinical and that delay can permit rapid extension of necrosis [3,6]. In this case, ultrasonography initially suggested epididymo-orchitis with funiculitis and pyocele, whereas the severity of the inflammatory response prompted further imaging. Computed tomography then demonstrated extensive peripenile and periscrotal inflammatory infiltration with multiple soft-tissue gas foci, strongly supporting a necrotizing infection. CT is useful for defining disease extent and detecting gas or deep fascial involvement, but imaging should not delay operative management when FG is clinically suspected [2,6].

The cornerstone of treatment is urgent surgical source control combined with broad-spectrum antimicrobial therapy and supportive care [2,6,10]. Debridement must remove all clearly devitalized tissue, and serial operative reassessment may be necessary because tissue necrosis can evolve after the first procedure. In our patient, emergency exploration revealed extensive purulent material and necrotic tissue; debridement was followed by open wound management and culture-directed antibiotics. This sequence is consistent with contemporary recommendations and with pediatric reports in which early surgery is central to survival and preservation of viable tissue [4-6].

Microbiologically, FG may be polymicrobial or monomicrobial. The organisms reported vary according to the portal of entry, underlying disease, prior antimicrobial exposure, and healthcare context [2,6]. In our case, intraoperative culture isolated ceftriaxone-resistant *K. pneumoniae*, leading to treatment with imipenem and amikacin according to susceptibility testing. This finding reinforces the importance of obtaining deep operative specimens rather than relying solely on empirical therapy, particularly in patients with repeated urinary instrumentation or previous healthcare exposure, in whom resistant Gram-negative organisms may be encountered.

HBOT was used only after emergency surgical debridement and antimicrobial treatment had been instituted. Its proposed benefit in necrotizing infection is biologically plausible: hyperoxia increases oxygen availability in hypoperfused tissues, supports oxygen-dependent neutrophil function, and may enhance fibroblast activity, collagen formation, angiogenesis, and wound repair [13,14]. These mechanisms are attractive in FG because thrombosis and edema impair local oxygen delivery. Nevertheless, HBOT cannot sterilize necrotic tissue or replace source control; its role is therefore strictly adjunctive.

The clinical evidence for HBOT in FG remains mixed and is dominated by adult observational data. Schneidewind et al. found substantial heterogeneity and emphasized the absence of high-quality randomized evidence [13]. In a 2022 meta-analysis of 10 observational studies, Raizandha et al. reported lower mortality with HBOT than with conventional treatment (odds ratio: 0.29; 95% CI: 0.12-0.69; p = 0.005), while hospital length of stay and number of debridements did not differ significantly [14]. A more recent meta-analysis similarly found an association between HBOT and reduced mortality but continued to identify uncertainty regarding treatment protocols, patient selection, and secondary outcomes [15]. These findings support consideration of HBOT in selected patients when immediately available, but they do not justify delaying debridement or transferring an unstable patient solely to obtain HBOT.

The pediatric evidence is considerably weaker. Contemporary pediatric reviews note that treatment recommendations are largely extrapolated from adult FG and broader necrotizing soft-tissue infection literature because pediatric cohorts are small and HBOT-specific data are sparse [6]. Recent pediatric case reports and series continue to demonstrate favorable outcomes with prompt antibiotics, debridement, and individualized wound management, but they do not establish the independent effect of HBOT [11,12,16]. Accordingly, the favorable evolution in our patient should be interpreted as the result of a multimodal strategy rather than as evidence of HBOT efficacy in isolation.

Wound management after infection control is another major component of care. Extensive debridement may leave substantial genital or perineal defects, and the timing of closure must balance eradication of infection against the benefits of earlier reconstruction. A recent pediatric report described successful closure following adequate initial debridement in a selected child [11]. In our patient, daily local care together with adjunctive HBOT was followed by progressive improvement, permitting surgical revision and closure of the scrotal wound. This individualized approach is consistent with the principle that reconstruction should be considered only after viable tissue and satisfactory infection control are established.

This case has several limitations. It is a single observation and cannot establish a causal benefit of HBOT. The relative contribution of HBOT cannot be separated from prompt debridement, culture-directed antimicrobial therapy, supportive care, and intensive wound management. In addition, pediatric HBOT protocols for FG are not standardized, limiting comparison across reports [6,13-15]. Despite these limitations, the case contributes to the limited pediatric literature by documenting the feasibility of incorporating HBOT into a multidisciplinary strategy after adequate surgical source control in an adolescent with significant urological comorbidity and a resistant Gram-negative infection.

## Conclusions

FG is a rare but potentially life-threatening condition in children that requires immediate recognition and aggressive multidisciplinary management. Pediatric patients may present with nonspecific scrotal or perineal inflammatory findings, particularly when underlying urological abnormalities are present.

Early surgical exploration and complete debridement, combined with appropriate antimicrobial therapy and meticulous wound care, remain the essential components of treatment. In selected patients, HBOT may be considered as an adjunctive therapy to optimize tissue oxygenation and support wound healing after adequate surgical source control.

Our case illustrates the potential feasibility of HBOT in the management of severe pediatric FG and highlights the importance of a multidisciplinary approach involving pediatricians, pediatric surgeons/urologists, infectious disease specialists, wound-care teams, and hyperbaric medicine specialists. Further pediatric studies are needed to determine the efficacy, optimal timing, treatment protocol, and patient selection criteria for HBOT in FG.
